# Orthodeoxia and its implications on awake-proning in COVID-19 pneumonia

**DOI:** 10.1186/s13054-021-03859-0

**Published:** 2021-12-16

**Authors:** Lorenzo Giosa, Didier Payen, Mattia Busana, Alessio Mattei, Luca Brazzi, Pietro Caironi

**Affiliations:** 1 Department of Surgical Sciences, University of Turin, Turin, Italy; 2 University of Paris, 7, Denis Diderot, Paris, France; 3 Department of Anesthesiology, University Medical Center Göttingen, Göttingen, Germany; 4 Department of Cardio-Thoracic Diseases, ‘Città della Salute e della Scienza’ University Hospital, Turin, Italy; 5 Department of Anaesthesia, Intensive Care and Emergency, ‘Città della salute e della Scienza’ University Hospital, Turin, Italy; 6 Department of Anesthesia and Critical Care, AOU S. Luigi Gonzaga, Turin, Italy; 7 Department of Oncology, University of Turin, Turin, Italy

Dear editor,

When caring for patients with respiratory failure, decubitus is a daily challenge. In the acute-respiratory-distress-syndrome (ARDS), seated and prone position increase lung volume and, consequently, oxygenation [1]. In COVID-19, however, gas-exchange is often independent of lung volume [2], and rather affected by perfusion dysregulation [3]. In similar settings, like the hepato-pulmonary syndrome (HPS), recumbency may revert hypoxemia [4]: this phenomenon goes under the name of orthodeoxia, and here we hypothesize its presence in COVID-19. Clinical implications might be relevant: recumbency is the state of lying horizontally at 0°, supine or prone. Awake-proning has already proven beneficial on oxygenation in spontaneously breathing patients with early COVID-19 pneumonia [5]. However, as a heritage from ARDS, these patients are usually seated or semi-recumbent, thereby the ventral decubitus is rarely compared to supination at 0°: the finding of orthodeoxia may lead to partially ascribe the oxygenation benefits of awake-proning [5] to recumbency rather than to the ventral decubitus itself.

At the University Hospital of Turin (Italy), following ethical approval (Città della Salute e della Scienza 00581/2020), we studied non-sedated COVID-19 patients requiring early (< 7 days) respiratory support with helmet continuous positive airway pressure (HCPAP) or high flow nasal cannula (HFNC). Concomitant pulmonary embolism and/or bacterial pneumonia represented exclusion criteria. After signing a written informed consent, participants were assigned to a random sequence of seated (trunk elevation > 60°, legs down at 45°), supine and prone position (both recumbent at 0°) during constant respiratory support as set by the attending physician. Blood gases, respiratory rate, dyspnea and discomfort, basic hemodynamics and, when available, cardiac output (CNAP®, CNSystems Medizintechnik GmbH) were assessed twenty minutes from each decubitus. A threshold of ≥ 20% increase in PaO_2_ defined supine responders (supine vs seated) and prone responders (prone vs supine). The primary outcome was the frequency of orthodeoxia (supine responders). R-3.5.2 was used for statistical computing: Wilcoxon test for median comparisons, Fisher exact test for contingency tables, two-sided *p* < 0.05 for significance.

After excluding 28 eligible patients (21 for pulmonary embolism, 7 for superimposed bacterial pneumonia), 30 were recruited in two months (February–March 2021); two declined to participate. Results and baseline characteristics of the 28 enrolled patients are summarized in Table 1. Orthodeoxia was detected in 14 (50%) of them, with a far higher PaO_2_ increase (31 [26–44] mmHg), than what normally required to define it (4 mmHg) [3]. Neither the starting decubitus (*p* = 0.33), nor the type of respiratory support (HCPAP or HFNC, *p* = 1.00) affected this result, and the stability of cardiac output from seated to supine minimizes the possibility that macro-hemodynamics played any significant role. A decrease in respiratory rate in the absence of dyspnea and discomfort was also associated with supination in our population. During proning, patients with and without orthodeoxia behaved similarly: respectively, 6 (46%) and 5 (36%) were prone responders (*p* = 0.70, median PaO_2_ increase 65[30–92] mmHg). This suggests that orthodeoxia cannot anticipate the response to proning, likely because of the unpredictable balance between perfusion redistribution and parenchymal reaeration in the ventral position [6]. However, the finding of orthodeoxia avoided overestimating the benefits of awake-pronation in 6 patients (22%) whose oxygenation improvement was due to lying recumbent at 0°, irrespective of prone or supine specifically (Fig. 1, green dots). Considering that the ventral decubitus was associated with discomfort, higher respiratory and heart rate, the decision to prone would be questionable in these patients.

**Fig. 1:**
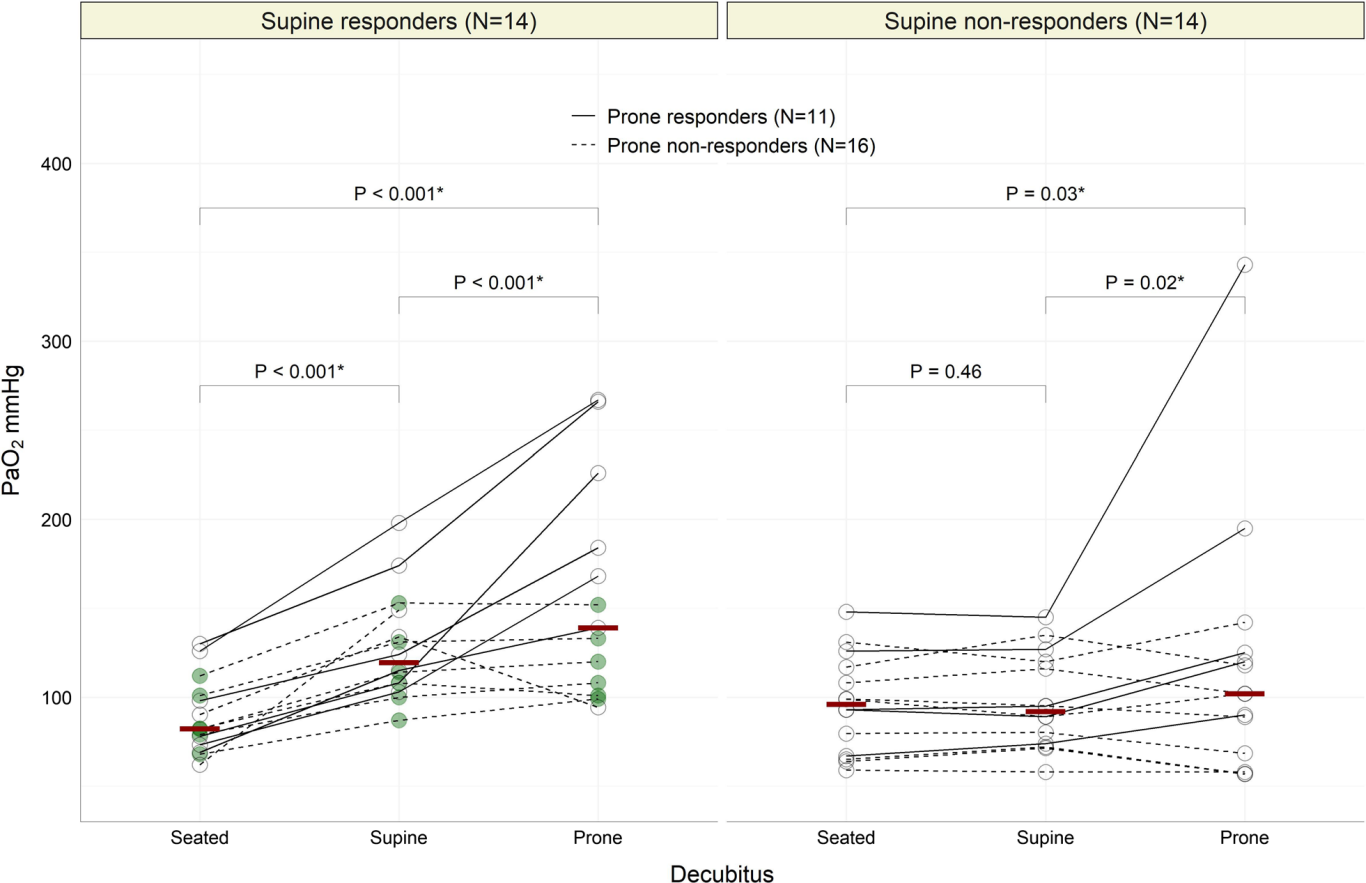
Individual Partial Pressure of Arterial Oxygen (PaO_2_) Variation in Supine responders (left) and Supine non-responders (right). In both groups solid lines represent prone responders, dashed lines prone non-responders (see Text for definitions). Red bars represent median PaO_2_ values in each decubitus, and *P* values (* when significant) refer to their comparisons. As shown, 14 patients (50%) were supine responders (median PaO_2_ increase from seated to supine: 31 [26–44] mmHg). Among these, one did not tolerate proning, six were prone responders (median PaO_2_ increase from supine to prone: 67 [60–92] mmHg) and seven were prone non-responders (one worsened oxygenation during proning, while the 6 patients highlighted by green dots benefit from recumbency irrespective of supine or prone position specifically). The remaining 14 patients (50%) were supine non-responders. Among these, 5 were prone responders (median PaO_2_ increase from supine to prone: 31 [30–68] mmHg), while in the 9 remaining subjects, PaO_2_ did not significantly change between supination and proning

In conclusion, orthodeoxia appears a common clinical feature of early COVID-19 pneumonia. This novel finding contributes to further distinguishing COVID-19 from other causes of ARDS [1, 2, 6], while reinforcing its advocated similarity with HPS [3, 4]. Additionally, detecting orthodeoxia may help avoid awake-pronation when oxygenation simply benefits from recumbency: in a pandemic scenario, this possibly relevant clinical implication would deserve confirmation by larger studies.

**Table 1:** Characteristics of Patients and Main Results

	SUPINE RESPONDERS			SUPINE NON-RESPONDERS		
**Baseline characteristics**						
No (%)	14 (50)			14 (50)		
Age, median (IQR)	66 (56–72)			66 (57–69)		
Sex, No (%)						
*Women*	1 (7.1)			5 (35.7)		
*Men*	13 (92.9)			9 (64.3)		
BMI, median (IQR)	27.5 (24.2–30.0)			28.3 (27.5–31.1)		
Current smokers, No (%)	1 (7)			0 (0)		
Arterial hypertension, No (%)	7 (50)			9 (64)		
Type 2 Diabetes Mellitus, No (%)	1 (7)			4 (29)		
SOFA score, median (IQR)	3 (3–3)			3 (2–3)		
*Disease course, median (IQR)*						
Days from diagnosis of infection	11.5(8–14)			9.5 (8–12)		
Days from hospital admission	3.5 (2–7)			3.5 (2–6)		
Days from respiratory support	2.5 (1–5)			2 (1–4)		
*Ventilatory settings*						
HCPAP, No (%)	11 (79)			11 (79)		
HFNC, No (%)	3 (21)			3 (21)		
FiO_2_, median (IQR)	0.5 (0.5–0.6)			0.5 (0.5–0.5)		
PEEP (if HCPAP), median (IQR)	10 (10–12)			10 (10–12)		
Flow (if HFNC), median (IQR)	40 (35–40)			35 (35–40)		

Protocol	Seated	Supine	Prone	Seated	Supine	Prone
Starting decubitus, no (%)	2 (14)	6 (43)	6 (43)	4 (29)	5 (36)	5 (36)
*Respiratory variables, median (IQR)*						
PaO_2_, mmHg	82.2 (73.2–101)	120 (108–149)*	139 (108–184)*	96 (67–117)	92 (74–120)	102 (68.5–125)*
PaO_2_/FiO_2_ ratio	152 (133–177)	224 (186–248)*	278 (198–336)*	192 (160–224)	186 (165–230)	204 (150–246)
PaCO_2_, mmHg	38 (35.9–39)	39.1 (38–43)*	37.8 (37–41)*	38.3 (34.1–40)	40.5 (35.8–42)*	39.8 (36–41)
Arterial pH	7.45 (7.44 -7.46)	7.43 (7.43 -7.45)*	7.44 (7.44 -7.46)	7.46 (7.44 -7.47)	7.45 (7.42 -7.47)*	7.44 (7.43 -7.46)
Respiratory rate, bpm	19 (17–22)	17 (15–18)*	19 (16–23)*	21.5 (18–24)	19 (16–22)*	18.5 (16–21)
*Subjective variables*						
Borg dyspnea scale, median (IQR)	0 (0–0)	0 (0–0)	0 (0–0)	0 (0–0)	0 (0–0)	0 (0–1)
Discomfort, no (%)	2 (14.3)	1 (7.1)	8 (57.1)*	0 (0)	0 (0)	5 (35.7)*
*Hemodynamics, median (IQR)*						
Cardiac index, L/min/m^2 a^	3 (3–3.2)	3.1 (3–3.3)	3.3 (3.3–3.5)*	2.8 (2.5–3.5)	2.9 (2.4–3.1)	3.1 (3–3.4)*
Stroke volume index, mL/m^2 a^	41 (39–42)	47 (38–48)*	46 (40–52)	39 (33–45)	50 (37–55)*	40 (37–51)
Pulse pressure, mmHg	51 (42–58)	55 (47–60)*	54 (48–70)	55 (46–78)	69 (53–90)*	71 (56- 81)
Heart rate, bpm	70 (69–77)	64 (60–71)*	68 (60–77)*	75 (67–86)	70 (60–76)*	76 (69–82)*

IQR, interquartile range; BMI, body mass index (weight in kilograms divided by the square of the height in meters); SOFA, sequential organ failure assessment; HCPAP, helmet continuous positive airway pressure; HFNC, high flow nasal cannula; FiO_2_, fraction of inspired oxygen; PEEP, positive end expiratory pressure; PaO_2_, partial pressure of oxygen; mmHg, millimeters of mercury; PaCO_2_, partial pressure of carbon dioxide; bpm, breaths (or beats) per minute

^a^Data available from 12 patients (5 supine responders and 7 supine non-responders) equipped with non-invasive advanced hemodynamic monitoring (CNAP®). Note that changes in stroke volume are paralleled by changes in pulse pressure (its surrogate) confirming the trend of cardiac output even in patients without advanced hemodynamic monitoring

^*^Significantly different (*p* < 0.05) with respect to the preceding decubitus in the table

## Data Availability

The dataset used and analysed for this study is available from the corresponding author upon reasonable request.

## References

[1] Mezidi M, Guérin C (2018). Effects of patient positioning on respiratory mechanics in mechanically ventilated ICU patients. Ann Transl Med.

[2] Coppola S, Chiumello D, Busana M, Giola E, Palermo P, Pozzi T (2021). Role of total lung stress on the progression of early COVID-19 pneumonia. Intensive Care Med.

[3] Reynolds AS, Lee AG, Renz J, DeSantis K, Liang J, Powell CA, Ventetuolo CE, Poor HD (2020). Pulmonary vascular dilatation detected by automated transcranial Doppler in COVID-19 pneumonia. Am J Respir Crit Care Med.

[4] Gómez FP, Martinez-Palli G, Barberà JA, Roca J, Navasa M, Rodriguez-Roisin R (2004). Gas exchange mechanism of orthodeoxia in hepatopulmonary syndrome. Hepatology.

[5] Coppo A, Bellani G, Winterton D, Di Pierro M, Soria A, Faverio P (2020). Feasibility and physiological effects of prone positioning in non-intubated patients with acute respiratory failure due to COVID-19 (PRON-COVID): a prospective cohort study. Lancet Respir Med.

[6] Rossi S, Palumbo MM, Sverzellati N, Busana M, Malchiodi L, Bresciani P (2021). Mechanisms of oxygenation responses to proning and recruitment in COVID-19 pneumonia. Intensive Care Med.

